# Robust Metformin Nanosystem Promotes Hair Growth in Androgenetic Alopecia

**DOI:** 10.34133/research.0780

**Published:** 2025-07-18

**Authors:** Qiuying Mai, Weisen Lin, Xiaoyu Qin, Guowang Cheng, Chen Wang, Guangtao Yu, Tongkai Chen

**Affiliations:** ^1^ Science and Technology Innovation Center, Guangzhou University of Chinese Medicine, Guangzhou 510405, China.; ^2^ Stomatological Hospital, School of Stomatology, Southern Medical University, Guangzhou 510280, China.

## Abstract

Androgenetic alopecia (AGA)—a condition characterized by hair loss due to aging, autoimmune responses, stress, and other factors—results in hair follicle (HF) shrinkage and dermal papilla cell apoptosis. So far, only minoxidil (MXD) and finasteride have been approved for AGA treatment. However, both drugs have serious side effects, including hypersensitivity and sexual dysfunction. Hence, novel treatment agents are required for AGA. Although metformin (Met) is primarily a diabetes drug, it is also known to promote hair growth. However, it shows low transdermal permeability, and the mechanisms underlying its therapeutic effects on AGA remain unclear. Two-dimensional black phosphorus nanosheets (BP NSs) have attracted attention as drug carriers due to their low cytotoxicity, good biocompatibility, and strong antioxidant capacity. However, they are unstable, prone to degradation, and unsuitable for transdermal drug delivery. Fortunately, this limitation can be addressed through modification strategies, such as polyethylene glycol (PEG) addition (PEGylation). Here, we PEGylated BP NSs to improve their stability and loaded them with Met to generate a transdermal system (BP-PEG-Met) for AGA treatment. Compared with topical MXD, BP-PEG-Met markedly promoted hair regeneration while inducing fewer side effects. BP-PEG-Met scavenged excessive reactive oxygen species in skin cells, reducing the oxidative stress around HFs. Moreover, it up-regulated the expression of vascular endothelial growth factor (VEGF) and platelet-endothelial cell adhesion molecule-1 (CD31) in dermal papillae, inducing angiogenesis around HFs and accelerating the hair cycle toward anagen. Overall, this BP-PEG-Met transdermal delivery system showed marked clinical potential as a multifunctional tool for treating alopecia and possibly managing other skin conditions.

## Introduction

Hair loss is a common condition and can adversely affect an individual’s psychological well-being [1]. The most prevalent type of hair loss is androgenetic alopecia (AGA) [2], which affects an estimated 50% and 80% of women and men, respectively [3]. The incidence of AGA is currently rising, and the age of AGA onset is decreasing steadily [4]. To date, only 2 drugs—minoxidil (MXD) and finasteride (FIN)—have been approved by the US Food and Drug Administration for AGA treatment. However, MXD can cause scalp itching, dandruff, hypertrichosis, and even transient hair loss in the early stages of treatment [5]. Meanwhile, reduced libido and sexual dysfunction are some common side effects of FIN treatment [6]. Therefore, new drugs are required to manage the growing challenge of AGA.

AGA is characterized by reduced hair follicle (HF) size and dermal papilla cell (DPC) apoptosis [7]. The cycle of hair growth, which involves the anagen, catagen, and telogen phases, is altered in AGA, contributing to HF miniaturization [8,9]. Although the mechanisms underlying AGA remain unclear, evidence suggests that the pathological microenvironment plays a key role in the disease process [10]. Notably, oxidative stress (OS) and vascular insufficiency are prominent features of the HF microenvironment in AGA [11]. OS, a core pathological feature of AGA, is caused by the excessive production of reactive oxygen species (ROS) [12]. Research shows that in AGA patients, OS levels are higher in bald areas than in nonbald areas, and OS increases the sensitivity of DPCs to androgens such as testosterone (TS) and its downstream product dihydrotestosterone (DHT). The continuous presence of DHT within the local environment stimulates hair papillae and accelerates the aging of DPCs [13]. In turn, these progeric DPCs secrete transforming growth factor β (TGF-β), which shortens anagen and prolongs telogen [14]. Furthermore, excess ROS blocks the development of HF stem cells (HFSCs) by inhibiting the Wnt/β-catenin axis, leading to HF miniaturization [15,16]. Vascular insufficiency also acts as a key pathological feature of the AGA HF microenvironment. Under normal circumstances, blood vessels supply oxygen and nutrients to DPCs for hair regeneration. However, TGF-β, secreted through paracrine mechanisms, promotes the apoptosis of vascular endothelial cells [17]. Furthermore, DHT reduces blood vessel formation around HFs by lowering vascular endothelial growth factor (VEGF) levels, leading to nutrient deficiency, which exacerbates AGA pathology [18]. Therefore, improving the pathological microenvironment of HFs by mitigating OS and vascular insufficiency could enable AGA management.

Black phosphorus nanosheets (BP NSs) have shown great potential in biosensing, tumor imaging, and drug delivery due to their unique characteristics, including superior drug-loading capacity, biocompatibility, and biodegradability [19,20]. The high drug-loading capacity of BP NSs primarily stems from their wrinkled, negatively charged surface and large surface area, which enable drug loading through coordination reactions and electrostatic interactions [21]. Additionally, BP NSs act as ROS scavengers and can reduce OS in tissues such as HFs [22]. However, BP NSs are prone to degradation under normal environmental conditions. The surface of BP NSs contains a lone pair of electrons that is prone to oxidation, leading to instability and limiting the application of BP NSs [23]. Fortunately, modifying negatively charged BP NSs with polyethylene glycol-amine (PEG-NH_2_) moieties carrying positive charges can enhance their stability [24] since PEG-NH_2_ captures the lone-pair electron.

Metformin (Met) is typically used for treating diabetes [25]. Interestingly, one study in a shaved mouse model showed that Met can also promote hair regrowth [26]. However, the transdermal penetration of Met is relatively limited [27]. Given that PEGylation can promote the transdermal permeation of BP NSs by expanding intercellular spaces through hydration [28], loading Met onto such complexes appears to be a feasible strategy for promoting its transdermal delivery. The guanidyl group of Met carries a delocalized positive charge, which can interact with the negative charge of BP NSs through electrostatic and hydrogen bonding [29]. Such interactions could also capture the lone-pair electrons of BP NSs, preventing carrier degradation. Thus, a PEGylated BP NSs-loaded Met drug delivery system (BP-PEG-Met) could be useful for the treatment of AGA.

**Fig. 1.**
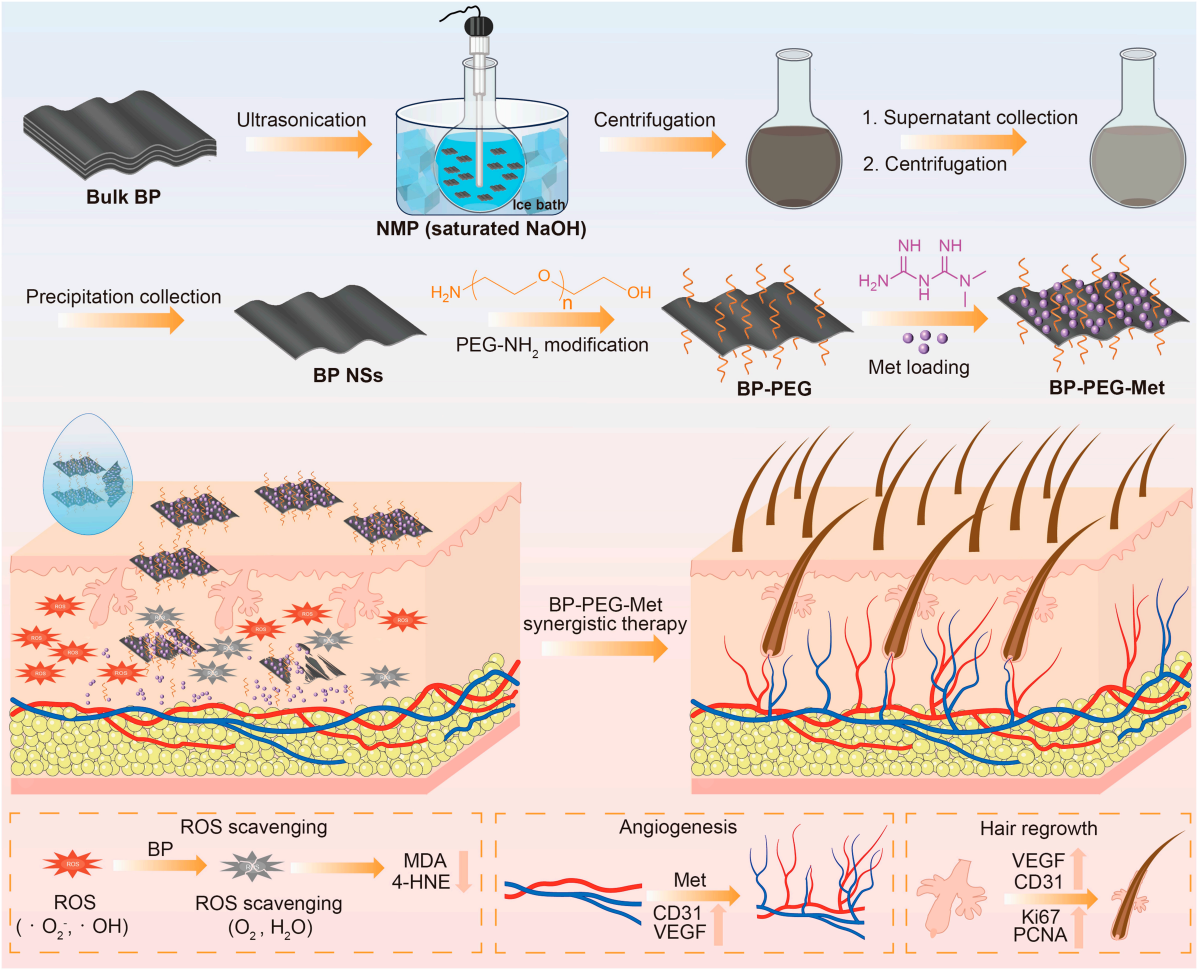
Schematic illustrating the construction of BP-PEG-Met and its therapeutic effects on AGA. BP-PEG-Met promotes hair regrowth by scavenging excess ROS in skin tissue, reducing oxidative stress in HFs, and up-regulating VEGF and CD31 to induce angiogenesis.

Based on these findings, we modified BP NSs with PEG to enhance their stability. Then, we loaded this complex with Met through electrostatic interactions to successfully construct a BP-PEG-Met drug-delivery system for AGA treatment (Fig. 1). In this study, a transdermal administration approach was employed to deliver BP NSs and Met to HFs. The BP-PEG-Met drug delivery system could alleviate OS in the HF microenvironment by reducing ROS levels. It could also up-regulate both VEGF and platelet-endothelial cell adhesion molecule-1 (CD31), thereby promoting angiogenesis in the dermal papilla and around HFs. Such dual regulation improved the HF microenvironment and stimulated hair regeneration, providing a novel approach for treating AGA.

## Results and Discussion

### Characterization and performance of BP NSs and BP-PEG-Met

**Fig. 2.**
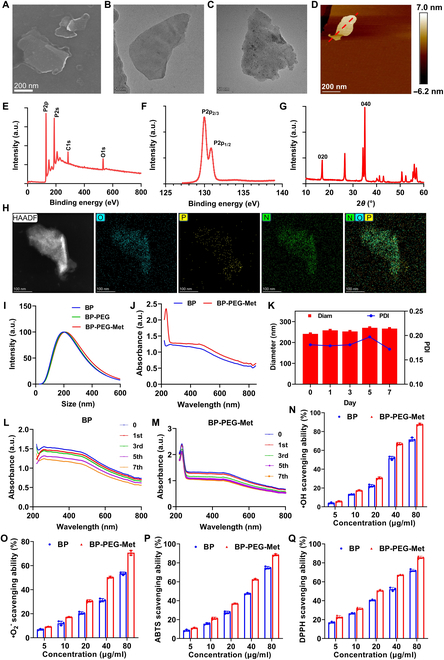
Characteristics and performance of BP NSs and BP-PEG-Met. (A) SEM and (B) TEM images of BP NSs. (C) TEM micrograph of BP-PEG-Met. (D) AFM image, (E) XPS analysis, (F) P2p spectrum, and (G) XRD analysis of BP NSs. (H) SEM micrograph and elemental map of BP-PEG-Met. (I) Particle sizes of BP NSs, BP-PEG, and BP-PEG-Met. (J) UV–Vis spectrograms of BP NSs and BP-PEG-Met. (K) DLS evaluation of BP-PEG-Met during 7 days of storage. Stability investigation (*n* = 3). (L to Q) UV–Vis spectroscopy analysis of BP NSs (L) and BP-PEG-Met (M) during 7 days of storage. (N) •OH, (O) •O_2_^−^, (P) ABTS, and (Q) DPPH scavenging ability of BP NSs and BP-PEG-Met (*n* = 3).

The surface morphology of BP NSs was evaluated using both scanning and transmission electron microscopy (SEM and TEM, respectively). As shown in Fig. 2A and B, the BP NSs exhibited a thin, transparent, and layered morphology and were approximately 200 nm in size. Atomic force microscopy (AFM) showed that their thickness was less than 7 nm (Fig. 2D and Fig. S1), consistent with that of phosphorene crystals containing a few layers.

The chemical composition of the BP NSs was assessed using x-ray photoelectron spectroscopy (XPS). As illustrated in Fig. 2E and F, 2 strong peaks were observed at 133.83 and 133.98 eV, corresponding to P2*p*^2/3^ and P2*p*^1/2^, respectively. Compared with the peaks seen in bulk BP, the BP NS peaks showed a slight red shift, indicating the successful exfoliation of ultrathin BP NSs from bulk BP. X-ray diffraction (XRD) analysis further confirmed the formation of ultrathin BP NSs, since characteristic peaks were detected at 16.7° and 34.7° (Fig. 2G). These findings were consistent with the reported literature [30].

Based on previous studies, BP NSs were modified with PEG-NH_2_ to enhance their stability [31]. Subsequently, Met was loaded onto PEGylated BP NSs (BP-PEG) via electrostatic interactions to prepare BP-PEG-Met. The surface morphology of BP-PEG-Met was characterized using TEM (Fig. 2C). In addition, elemental mapping revealed that the P and O atoms in BP-PEG-Met were derived from BP, while the N atoms were derived from Met (Fig. 2H). Dynamic light scattering (DLS) experiments demonstrated that the hydrodynamic size of BP-PEG-Met (226 nm) was markedly greater than that of BP NSs (193 nm) and BP-PEG (218 nm) (Fig. 2I). Meanwhile, the zeta potential of BP-PEG-Met was also significantly altered. Both these changes further verified the successful loading of Met onto BP-PEG (Fig. S2). Finally, drug loading capacity was determined using ultraviolet–visible (UV–Vis) spectroscopy. As illustrated in Fig. 2J, BP-PEG-Met showed characteristic absorption peaks at 233 nm, corresponding to Met. This indicated that Met was successfully loaded onto BP NSs. The Raman spectra further verified that PEG and Met modifications affected the molecular vibration characteristics of BP NSs (Fig. S3). Furthermore, BP-PEG-Met exhibited a high drug-loading rate, with the drug-loading efficiency reaching 83.87% ± 3.94%. Compared with other Met-loaded carriers (Table S1), BP-PEG-Met showed a superior drug loading rate.

BP NSs must exhibit excellent stability to efficiently deliver drug molecules and exert antioxidant effects. Therefore, DLS was used to assess the hydrodynamic size and polydispersity index (PDI) of BP-PEG-Met during 7 days of storage. As shown in Fig. 2K, neither parameter changed significantly during storage, indicating that BP-PEG-Met possessed good stability. Additionally, the absorption peaks of BP-PEG-Met (Fig. 2M) declined more slowly during storage than those of BP NSs (Fig. 2L). This was attributed to the reduced exposure of BP NSs to oxygen and water after PEGylation, which effectively inhibited oxidation-induced degradation [32]. These findings demonstrated that BP-PEG-Met had good stability and could be suitable for drug delivery.

In vitro drug release affects drug absorption, safety, efficacy, and stability in vivo. Thus, it is an important quality control index for nanomedicines. As shown in Fig. S4, Met alone was completely released within 4 h. In contrast, BP-PEG-Met required more than 12 h to achieve 90% cumulative Met release. The drug release behavior of BP-PEG-Met largely followed first-order kinetics (Table S2). This was likely because Met was loaded onto BP NSs through electrostatic interactions [29], which enabled sustained drug release.

Finally, the permeation of topical Met and BP-PEG-Met was evaluated using the diffusion cell apparatus (Fig. S5). After 2 h, no significant difference in permeation was observed between the 2 formulations. However, the cumulative permeation of BP-PEG-Met (390.55 ± 34.27 μg/cm^2^) was significantly higher than that of Met (305.05 ± 0.19 μg/cm^2^) after 8 h. Drugs typically permeate the skin through the transcellular pathway, the accessory pathway, and the inter-cellular pathway [33,34]. Among these, the transcellular pathway is the most direct and fastest mode for drug permeation across the skin barrier. Nanomaterials such as BP NSs always cross the skin barrier through the transcellular pathway [35]. Our findings suggested that the BP-PEG-Met drug delivery system entered the skin through the transcellular pathway, thereby increasing Met permeation in vitro. Meanwhile, as a hydrophilic drug, Met was more prone to uptake through the inter-cellular pathway [36], resulting in relatively lower in vitro skin penetration. This also explained why BP-PEG-Met treatment showed a higher efficiency than topical Met application. Notably, our cumulative permeation curves indicated that the drug release behavior of BP-PEG-Met largely followed first-order kinetics (Table S3). After 24 h, the skin retention values of topical Met and BP-PEG-Met were 27.84 ± 0.02 μg and 35.06 ± 0.07 μg, respectively, indicating that BP-PEG-Met improved the retention of Met in the skin (Fig. S6). Moreover, BP-PEG-Met provided better Met delivery than previously reported formulations [37]. Collectively, the permeation assays indicated that BP-PEG-Met promotes both the permeation and retention of Met in the skin.

Functionally, OS causes marked damage to HFs, resulting in HF miniaturization and DPC apoptosis [38]. Eliminating excessive ROS may be a potential strategy to treat AGA. Thus, the antioxidant activities of BP NSs and BP-PEG-Met against various types of ROS, including •OH, •O_2_^−^, 2,2′-azino-bis(3-ethylbenzothiazoline-6-sulfonic acid) (ABTS), and 1,1-diphenyl-2-picrylhydrazyl (DPPH), were evaluated in this study. As shown in Fig. 2N to Q, both BP NSs and BP-PEG-Met displayed concentration-dependent ROS scavenging. Moreover, BP-PEG-Met concentrations of 80 μg/ml achieved more than 90% clearance against all types of ROS. Compared with BP NSs, BP-PEG-Met exhibited stronger antioxidative capacity. This enhanced antioxidative capacity was attributed to the combined antioxidant properties of BP NSs and Met, which resulted in synergistic effects [39]. Therefore, BP-PEG-Met showed potential as an efficient antioxidant for AGA treatment.

### In vitro safety evaluation and uptake of BP NSs by human skin fibroblast cells

The cytotoxicity profiles of BP NSs, Met, and BP-PEG-Met in human skin fibroblast cells (HSFCs) were evaluated. Flow cytometry analysis illustrated that BP NSs had desirable biocompatibility at concentrations of 1.25 to 20 μg/ml (Fig. S7). As shown in Figs. S8 and S9, Cell Counting Kit-8 (CCK-8) assays revealed that Met and BP-PEG-Met exerted no cytotoxicity within the tested concentration range. The cell viability in the BP-PEG-Met group remained above 85% across all tested concentrations. These results were confirmed using the live/dead assay, indicating the good biocompatibility of BP NSs, Met, and BP-PEG-Met (Fig. S10). Considering both efficacy and safety, a BP-PEG-Met concentration of 20 μg/ml was selected for subsequent experiments.

**Fig. 3.**
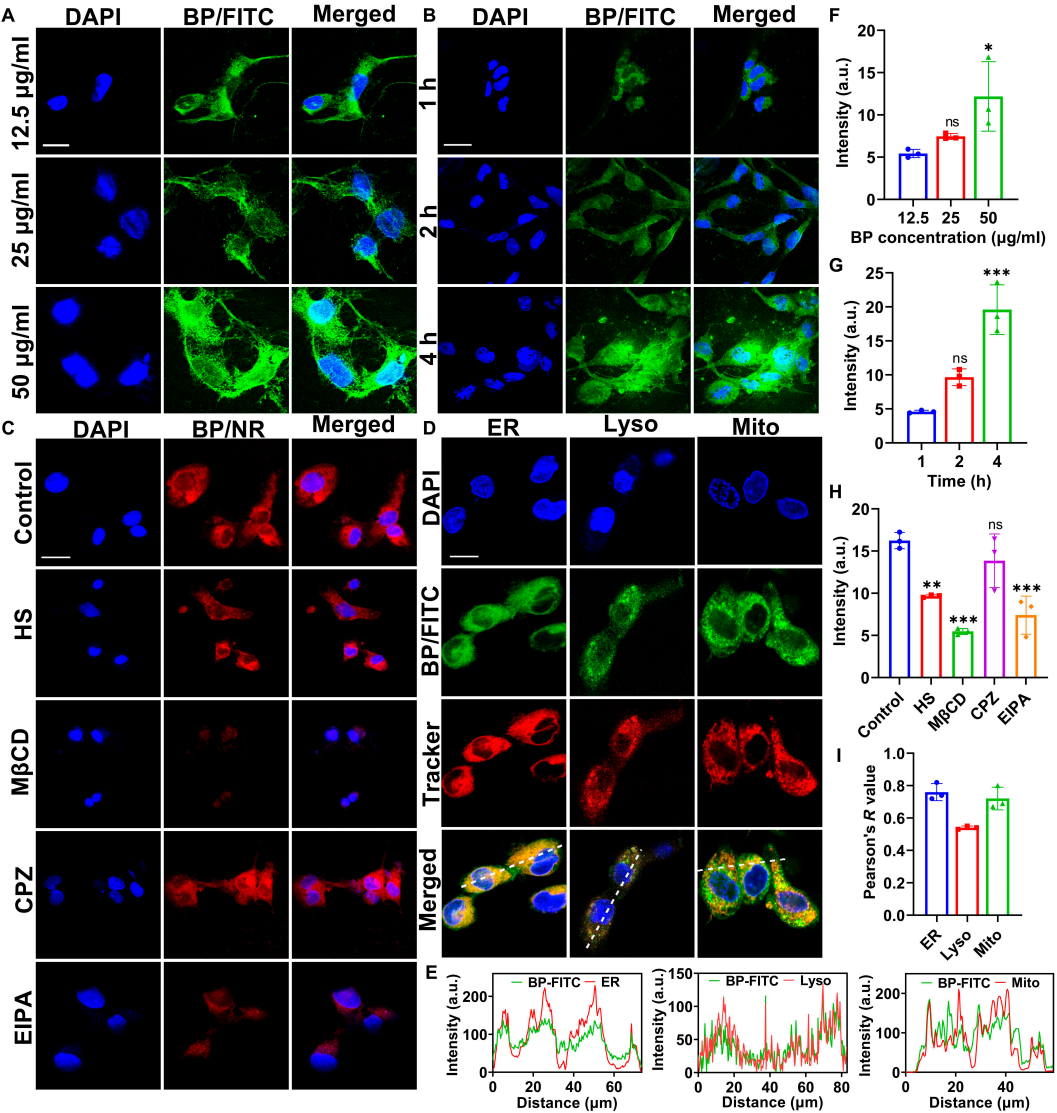
Cellular uptake and distribution of BP NSs. (A) Confocal images depicting the concentration-dependent and (B) time-dependent cellular uptake of BP NSs. Scale bar = 25 μm. (C) Confocal images showing the effects of various inhibitors on the endocytosis of BP-FITC in HFSCs. Scale bar = 25 μm. (D) Colocalization of BP-FITC with ER-Tracker, Lyso-Tracker, and Mito-Tracker in HFSCs. Scale bar = 20 μm. (E) Fluorescence intensity profiles in the cells along the white lines in (D). (F) Quantification of fluorescence intensity in (A) (*n* = 3); ns, no significant difference (*P* > 0.05), *P < 0.05 vs. the 12.5 μg/ml group. (G) Quantification of fluorescence intensity in (B) (*n* = 3); ns, no significant difference (*P* > 0.05), ***P < 0.001 vs. the 1-h group. (H) Quantification of fluorescence intensity in (C) (*n* = 3); ns, no significant difference (*P* > 0.05), **P < 0.01, ***P < 0.001 vs. the control group. (I) Correlation between the fluorescence intensity of BP-FITC and various organelle trackers

The cellular uptake in HSFCs was examined after treatment with varying concentrations of BP NSs. In these assays, a concentration-dependent uptake pattern was observed. As the concentration of BP NSs increased, the fluorescence signal in HSFCs became stronger (Fig. 3A). Moreover, when BP NSs were incubated with HSFCs for different durations, the fluorescence signal became stronger as incubation was prolonged. This showed that the uptake of BP NSs was also time-dependent (Fig. 3B). The quantitative analysis of fluorescence intensity provided similar results, demonstrating that the endocytosis of BP NSs was both concentration- and time-dependent (Fig. 3F and G).

A detailed understanding of cellular uptake pathways is crucial for elucidating drug action mechanisms. Therefore, Nile red (NR)-labeled BP NSs (BP-NR) were used to explore the endocytosis mechanisms and intracellular trafficking profiles of the prepared nanocarriers. Different endocytosis pathways were blocked using inhibitors to determine the mechanism underlying the uptake of BP NSs in HFSCs. Untreated cells incubated with BP NSs were used as controls, and their fluorescence intensities were normalized to 100%. As shown in Fig. 3C and H, after treatment with various inhibitors, the fluorescence intensity of BP-NR in HSFCs decreased. In particular, after treatment with methyl-β-cyclodextrin and ethylisopropylamiloride, the fluorescence intensity of BP-NR in HSFCs decreased significantly. These results indicated that the uptake of BP-NR by HSFCs involved multiple endocytic mechanisms. BP-NR primarily entered HSFCs through caveolin-mediated endocytosis and macropinocytosis, with clathrin-mediated endocytosis playing a relatively limited role, consistent with previous reports [40].

Lysosomes contain a variety of acidic hydrolases and act as intracellular scavengers. They not only degrade senescent and damaged intracellular organelles but also eliminate invading pathogens and remove metabolic waste [41]. Meanwhile, the endoplasmic reticulum (ER) is associated with protein production, lipid-related functions, calcium ion storage, and endocytic transport [42]. Additionally, mitochondria are unique organelles responsible for, among other functions, adenosine triphosphate (ATP) generation and lipid and carbohydrate metabolism [43]. They maintain calcium, copper, and iron homeostasis in cells and act as signal convergence points for several signaling pathways [44]. To track the intracellular fates of BP NSs following HSFC uptake, a confocal laser scanning microscope (CLSM) was utilized in this study, and colocalization analysis was performed. Specifically, the intracellular trafficking of fluorescein isothiocyanate (FITC)-labeled BP NSs (BP-FITC) was monitored. Positively charged FITC-NH_2_ was loaded onto negatively charged BP NSs via electrostatic adsorption. The ER, lysosomes, and mitochondria were stained using specific trackers, and the degree of fluorescence overlap between BP-FITC and these organelles was determined based on Pearson’s correlation coefficients. As shown in Fig. 3D and E, BP-FITC showed different degrees of fluorescence overlap with the ER, lysosomes, and mitochondria. This indicated that BP-FITC was distributed to all these organelles after cellular internalization. Cellular uptake assays showed that BP NSs entered cells through the endocytic pathway, indicating that they were most likely to enter lysosomes. However, BP NSs demonstrated greater colocalization with the ER and mitochondria (Fig. 3I). This suggested that only some of the BP NSs were phagocytosed, while the rest could escape lysosomes, likely owing to the “proton-sponge effect”. During the gradual acidification of lysosomes, negatively charged BP NSs could bind to protons (H^+^) and generate phosphates. This could increase osmotic pressure and cause water influx, leading to lysosomal swelling and rupture [45,46]. Subsequently, BP NSs could be released into the cytoplasm, thus achieving lysosomal escape. The observed intracellular distribution of BP NSs could enhance their capacity to alleviate cellular OS. Specifically, the accumulation of BP NSs in mitochondria could directly inhibit ROS production [47], regulate the mitochondrial membrane potential, and thereby promote ATP production [48,49]. Meanwhile, the accumulation of BP NSs in the ER could promote their transport to the cell periphery through intracellular transport pathways. These BP NSs could be released into the extracellular environment, facilitating uptake by neighboring cells [19,50,51].

In summary, the results showed that BP NSs mainly entered HSFCs through caveolin-mediated endocytosis and macropinocytosis. After entering HSFCs, the BP NSs were mainly distributed to the ER and mitochondria. Furthermore, BP NSs showed limited distribution in lysosomes, which reduced the probability of their intracellular degradation.

### Cytoprotective effects of BP-PEG-Met

**Fig. 4.**
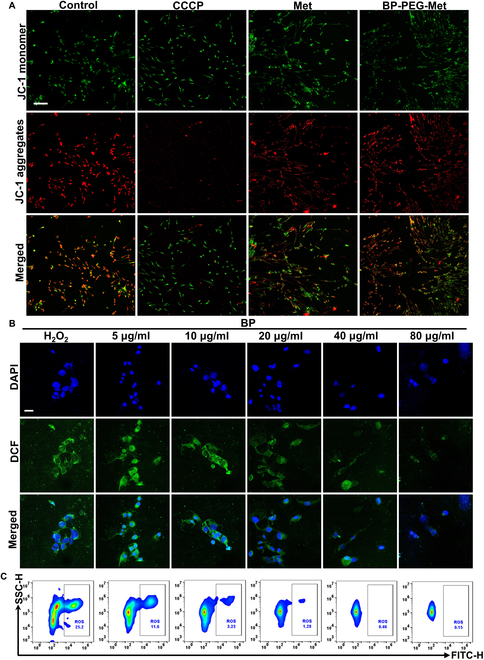
Cellular assays. (A) Mitochondrial membrane potential of HFSCs before and after treatment with BP NSs, Met, and BP-PEG-Met. Scale bar = 100 μm. (B) Fluorescence images showing the antioxidant capacity of HSFCs after treatment with different amounts of BP NSs. Scale bar = 100 μm. (C) Fluorescence intensity after treatment with different amounts of BP NSs (corresponding to B) quantified via flow cytometry.

Mitochondria are important sites of aerobic respiration. ATP is produced during mitochondrial oxidative phosphorylation when protons flow through ATP synthase along the electrochemical gradient [52]. Therefore, the mitochondrial membrane potential serves as a critical biophysical indicator of mitochondrial function. However, damage to the respiratory chain hinders electron transfer, leading to the generation of an excessive amount of ROS. This reduces the mitochondrial membrane potential, which further exacerbates the damage to the mitochondrial respiratory chain and increases OS, ultimately causing cellular dysfunction [53]. In this study, the mitochondrial membrane potential was determined to examine the protective effects of BP-PEG-Met in HSFCs. As illustrated in Fig. 4A and Fig. S11, in normal HSFCs, the presence of intense red fluorescence indicated a high mitochondrial membrane potential. After treatment with Met alone, the red fluorescence weakened while the green fluorescence intensified. This indicated that Met significantly decreased the mitochondrial membrane potential of HSFCs, likely by inhibiting Complex 1 in the mitochondrial respiratory chain [54]. However, compared with cells treated with carbonyl cyanide m-chlorophenylhydrazone (an oxidative phosphorylation inhibitor that disrupts the proton gradient and inhibits ATP synthesis, used to induce mitochondrial dysfunction) and Met, HSFCs treated with BP-PEG-Met exhibited weaker green fluorescence and stronger red fluorescence, suggesting that BP-PEG-Met exerted a protective effect on HSFCs. This could be attributed to the antioxidant effect of BP NSs and the consequent reduction in intracellular ROS levels [55]. The ROS-scavenging activity of BP NSs was assessed by measuring H_2_O_2_ levels after BP NSs treatment. The BP NSs-treated groups showed a concentration-dependent reduction in 2′,7′-dichlorofluorescin (DCF) fluorescence intensity (Fig. 4B), indicating that BP NSs could alleviate the OS induced by H_2_O_2_. Flow cytometry results validated these findings, demonstrating that BP NSs could protect HSFCs against OS (Fig. 4C). In addition, CCK-8 cell viability assays further validated these findings (Fig. S12).

### Effects of BP-PEG-Met on hair regeneration

**Fig. 5.**
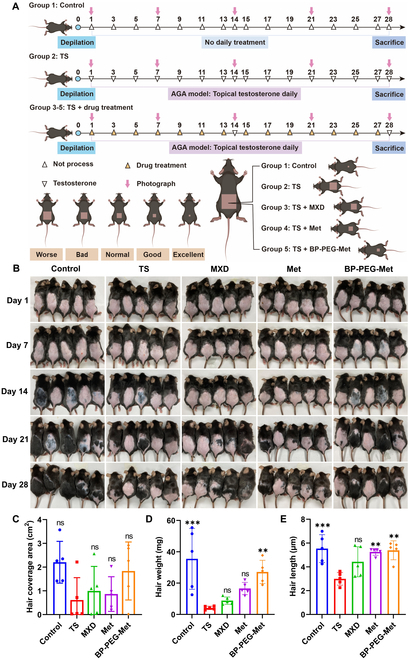
Regrowth of hair in a mouse model of AGA. (A) Schematic showing the establishment of the AGA model and the treatment protocols. Control mice received no treatment, while model mice were only treated with TS in the depilated areas. Met- and BP-PEG-Met-treated mice received the respective treatments on alternate days along with the daily topical application of TS. MXD mice received topical 5% MXD on alternate days during the 28-day period, along with daily TS application. (B) Representative images of hair regrowth in different groups on days 1, 7, 14, 21, and 28 following depilation. (C to E) Quantification of hair coverage (C), hair weight (D), and hair length (E) (*n* = 5). ns, no significant difference (*P* > 0.05), **P < 0.01, ***P < 0.001 vs. the model (TS) group.

To evaluate the hair regrowth efficacy of BP-PEG-Met, a mouse model of AGA was developed in C57BL/6 mice, which are commonly used to study hair regrowth. Figure 5A shows the treatment schedule. Specifically, the dorsal side of each mouse’s body was depilated, and TS was applied topically for 28 consecutive days. Subsequently, mice in the treatment groups received 5% MXD, Met, and BP-PEG-Met topically every other day.

In mice, the skin color on the dorsal side corresponds to the phase of hair growth. At 7 weeks of age, the dorsal skin of mice is typically pink, and this color is associated with telogen. As anagen begins, the skin darkens due to the activation of melanocytes [56]. In this study, photographs taken at different time points showed no evidence of regrowth in most of the model mice until day 28 (Fig. 5B), demonstrating the successful establishment of the AGA model. However, BP-PEG-Met treatment resulted in considerable hair regrowth and coverage. By day 7, the skin on the dorsal side appeared darker in the BP-PEG-Met group (Fig. 5B), indicating the initiation of anagen. This early transition was similar to that seen in control mice. Although the hair coverage area in the BP-PEG-Met group (Fig. 5C) was not significantly different from that in the other treatment groups, the skin color shift from pink to gray indicated earlier anagen phase entry. In contrast, the skin color in the model group remained pink, indicating a significant delay in the re-entry of HFs into the anagen phase. Importantly, the weight of regenerated hair (Fig. 5D) and hair length (Fig. 5E) were both significantly higher in the BP-PEG-Met animals. These results demonstrated that BP-PEG-Met promoted the entry of HFs into anagen and thus enhanced hair regeneration in the AGA mouse model.

### Histological analysis of mouse hair regrowth

**Fig. 6.**
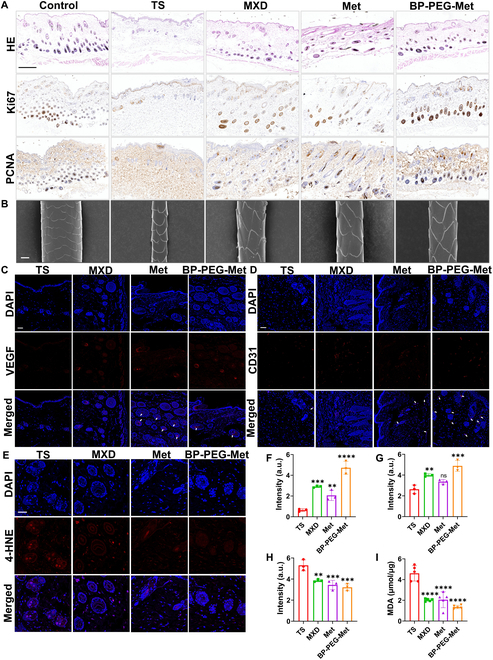
Histological analysis of depilated mouse skin and in vivo evaluation of the remodeling of the perifollicular microenvironment. (A) H&E staining and Ki67 and PCNA immunohistochemical staining of the dorsal skin tissues of mice on day 28 after depilation. Scale bar = 200 μm. (B) SEM images of hair regeneration on day 28. Scale bar =10 μm. (C) Fluorescence image showing VEGF expression in skin tissue. The arrows in the merged figure indicate the increase in VEGF expression. Scale bar = 50 μm. (D) Fluorescence image showing CD31 expression in skin tissues. The arrows in the merged figure indicate the increase in CD31 expression. Scale bar = 50 μm. (E) Fluorescence image showing 4-HNE levels in skin tissues. Scale bar = 50 μm. (F to H) Quantification of VEGF (F), CD31 (G), and 4-HNE (H) fluorescence in skin tissues (*n* = 3). (I) MDA content in skin tissues on day 28 (*n* = 5). ns, no significant difference (*P* > 0.05), **P < 0.01, ***P < 0.001, ****P < 0.0001 vs. the model (TS) group.

Hematoxylin and eosin (H&E) staining showed that on day 28 after depilation, mice treated with Met, MXD, and BP-PEG-Met exhibited significantly higher HF density than those treated with TS alone. Additionally, some HFs in these treatment groups showed increased volume and melanin production (Fig. 6A), indicating entry into the anagen phase. The findings suggested that treatment with Met, MXD, and BP-PEG-Met could promote hair growth in mice. The TS group showed the lowest number of HFs, with miniaturized HFs indicative of the telogen phase. Meanwhile, the number of HFs was the highest in BP-PEG-Met-treated mice, exceeding that in the MXD group. The volume of HFs was also increased in the BP-PEG-Met group, consistent with the anagen phase of hair growth (Fig. S13).

Antigen Kiel 67 (Ki67) and proliferating cell nuclear antigen (PCNA), as markers of cell proliferation, are up-regulated when HFs enter the anagen phase [57]. To more comprehensively confirm that BP-PEG-Met promotes HF cell proliferation, we evaluated Ki67 and PCNA expression in dermal papillae using immunohistochemical staining. Immunohistochemical analysis showed that Ki67 and PCNA levels were low in the TS group, indicating that these HFs were in the telogen phase. In contrast, Ki67 levels in the dermal papillae were high in the BP-PEG-Met group, indicating that the HFs were in anagen, showing active cellular proliferation. Moreover, the Ki67 levels in this group were similar to those in the control group. Both the MXD and Met groups also showed Ki67 and PCNA up-regulation, but not as much as the BP-PEG-Met group (Fig. 6A). These findings showed that BP-PEG-Met can induce the proliferation of HF matrix cells and promote the transition from telogen to anagen, thereby inducing hair regeneration. Furthermore, SEM analyses revealed that the new hair in TS-treated mice was thinner and had fewer scales, while hair in the BP-PEG-Met, Met, and MXD mice was thicker with complete scales (Fig. 6B). Although Met and MXD also enhanced hair regeneration, the highest quality of newly generated hair was observed in the BP-PEG-Met group.

### In vivo evaluation of remodeling around vesicles

Angiogenesis is important in the cycle of HF growth since it provides nutrition and activates hair growth. In the early stages of AGA-related HF miniaturization, the vasculature in the corresponding dermal papillae decreases [58]. Therefore, angiogenesis around dermal papillae and HFs is necessary for promoting hair growth [17]. Notably, VEGF is known to promote vascular permeability and angiogenesis [59]. Thus, we evaluated in vivo angiogenesis in the different groups of mice by detecting VEGF through immunofluorescence assays. Interestingly, minimal VEGF expression was detected around the HFs in the TS mice. Similarly, low levels of VEGF were also observed in the Met group. However, VEGF expression around the HFs and in the dermal papillae was significantly increased after BP-PEG-Met treatment (Fig. 6C and F), indicating that BP-PEG-Met enhanced VEGF expression in DPCs to induce hair regeneration.

VEGF can induce the growth of vascular endothelial cells, which express the marker CD31 [60]. Therefore, we also evaluated the expression of CD31 through immunofluorescence assays. CD31 expression around HFs and in dermal papillae was fairly limited in the TS and Met groups. However, CD31 expression at these sites increased considerably in the BP-PEG-Met group (Fig. 6D and G). This was consistent with the trends of VEGF expression, indicating that BP-PEG-Met promotes angiogenesis in the dermal papillae by up-regulating VEGF and CD31. Given that angiogenesis promotes anagen induction while also increasing the diameter of the dermal papillae and the length of the hair shaft, these findings demonstrated the potential therapeutic benefits of BP-PEG-Met [61].

OS allows androgens to act on dermal papillae, generating DHT through type II 5α-reductase, which leads to the apoptosis of DPCs [62]. Excessive ROS can disrupt HFs, promoting the occurrence and development of AGA [63]. Therefore, we speculated that the antioxidant effect of BP-PEG-Met would be important for protecting the HFs and promoting hair regeneration. The accumulation of ROS in the skin promotes lipid peroxidation, generating 4-hydroxy-2-nonenal (4-HNE), which can serve as a marker of oxidative damage [64]. In this study, high levels of 4-HNE were observed in the areas around HFs and the dermal papillae in the TS group, indicating excess ROS generation. In contrast, the decreased expression of 4-HNE in the Met and BP-PEG-Met groups reflected the antioxidant capacity of these treatment agents. The BP-PEG-Met group showed the most significant reduction in 4-HNE levels. This indicated that BP NSs had antioxidant capacity and could alleviate the OS around HFs (Fig. 6E and H).

Elevated levels of malondialdehyde (MDA), associated with lipid peroxidation, also act as an OS indicator in AGA patients [65]. Hence, we also measured the MDA content in skin tissues to validate the antioxidant activity of BP-PEG-Met (Fig. 6I). The MDA content was markedly lower in the Met and BP-PEG-Met groups than in the TS group. Evidence shows that Met inhibits mitochondrial complex I by activating the AMP-activated protein kinase–Forkhead box O3 (AMPK-FOXO3) pathway and thereby reduces ROS production [66]. In this study, the MDA reduction trend was more obvious in the BP-PEG-Met group. The ability of BP NSs to enhance the ROS-scavenging capacity of Met was confirmed by the immunofluorescence assays. The findings showed that BP-PEG-Met possessed good ROS scavenging effects in vivo, could mitigate oxidative damage in HFs, and could thereby remodel the HFs environment.

Studies have shown that OS can impair the vascular endothelium [67]. By scavenging ROS around HFs, BP-PEG-Met could promote angiogenesis within dermal papillae. Through dual regulation and the attenuation of HF disruption, BP-PEG-Met could induce the transition from telogen to anagen.

### Evaluation of in vivo biocompatibility

Given the high frequency of administration and the large number of doses, there was a potential risk of systemic toxicity due to the cumulative absorption of BP-PEG-Met. Therefore, the in vivo safety of BP-PEG-Met was examined. Mice were weighed every other day during the 28-day treatment period. Notably, the body weights of all mice increased over time, while their dietary habits and other activities remained normal. No significant differences in body weights were detected between the BP-PEG-Met and control groups, indicating that the treatment dose was safe and did not cause any health problems (Fig. S14).

**Fig. 7.**
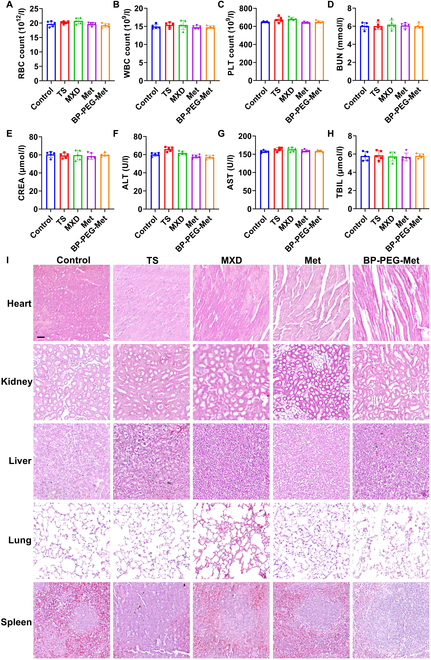
Evaluation of in vivo biocompatibility. (A) RBC counts. (B) WBC counts. (C) PLT counts. (D) BUN levels. (E) Serum CREA levels. (F) Serum ALT levels. (G) Serum AST levels. (H) Serum TBIL levels. (I) H&E staining of heart, liver, spleen, lung, and kidney tissues obtained from mice on day 28. Scale bar = 200 μm.

Hematological analysis was also performed to evaluate potential systemic toxicity. Specifically, the counts of red blood cells (RBCs), white blood cells (WBCs), and platelets (PLT) were evaluated. Notably, all relevant indicators remained within the normal range across all treatment groups (Fig. 7A to C). Further blood biochemical analyses of liver and kidney function (blood urea nitrogen [BUN], creatinine [CREA], alanine aminotransferase [ALT], aspartate aminotransferase [AST], and total bilirubin [TBIL]) revealed no abnormalities in these parameters in any of the treatment groups (Fig. 7D to H). This indicated that the BP-PEG-Met drug delivery system had good biological safety and did not cause functional hepatic or renal damage in mice.

Finally, the main organs of the mice were histologically analyzed for pathological changes using H&E staining (Fig. 7I). The myocardial cells in each group were intact, and the morphology of liver cells was normal, without any inflammation or fibrosis. Additionally, there was no inflammatory reaction in the spleen and lungs, and no inflammatory infiltration or glomerular atrophy was observed in the kidneys. Given the good in vivo biocompatibility of BP-PEG-Met and the growing interest in BP-based biomaterials, the BP-PEG-Met drug delivery system appeared suitable for AGA therapy.

## Conclusion

In this study, we prepared BP NSs using liquid-phase exfoliation and increased their stability through PEG-NH_2_ modification. Subsequently, we loaded modified BP NSs with Met via electrostatic interactions to successfully construct the BP-PEG-Met drug delivery system. Our in vitro experiments demonstrated that the antioxidant capacity of the prepared BP-PEG-Met system was superior to that of unmodified BP NSs, and BP-PEG-Met exerted no cytotoxicity in HFSCs. Importantly, the cellular uptake of BP NSs was both concentration- and time-dependent, and caveolin-mediated endocytosis and macropinocytosis were found to play a major role in the uptake of BP NSs. Subsequently, in vivo experiments showed that compared with topical MXD, BP-PEG-Met was more effective at scavenging ROS and could simultaneously up-regulate CD31 and VEGF to promote angiogenesis, thus improving the HF microenvironment through dual regulation to promote hair regrowth. Moreover, the BP-PEG-Met system exhibited good biosafety and did not cause skin damage. Collectively, the results demonstrated that the BP-PEG-Met drug delivery system constructed in this study is simple to prepare, can effectively induce hair regrowth, and exhibits high biosafety. Thus, it holds marked promise as a potential treatment agent for the management of AGA.

## Materials and Methods

Detailed information regarding the materials used, preparation and characterization methods, in vitro drug release assays, transdermal and skin retention analysis, and antioxidant activity assays of BP NSs and BP-PEG-Met, as well as cellular experiments, the in vivo treatment of AGA, evaluation of hair quality, histology, immunofluorescence staining, MDA assays, and statistical analyses are provided in Sections S1 to S14 of the Supplementary Materials.

## Data Availability

All data used to support the findings in the paper and the Supplementary Materials are available from the corresponding authors upon reasonable request.

## Supplementary Materials

Supplementary 1 Sections S1 to S14 Figs. S1 to S14 Tables S1 to S3
