# Supplementary material for: Robust Metformin Nanosystem Promotes Hair Growth in Androgenetic Alopecia
Source: Research (Wash D C). 2025 Jul 18;8:0780. doi: 10.34133/research.0780 (PMC12271744; doi:10.34133/research.0780)
Supplement: Supplementary 1 — Sections S1 to S14 Figs. S1 to S14 Tables S1 to S3 [file research.0780.f1.docx]

**Supporting Information**

**Robust Metformin Nanosystem Promotes Hair Growth in Androgenetic Alopecia**

Qiuying Mai^1, #^, Weisen Lin^1, #^, Xiaoyu Qin^1^, Guowang Cheng^1^, Chen Wang^1^, Guangtao Yu^2,^ *, Tongkai Chen^1,^ *

*^1^ Science and Technology Innovation Center, Guangzhou University of Chinese Medicine, Guangzhou 510405, China*

*^2^ Stomatological Hospital, School of Stomatology, Southern Medical University Guangzhou 510280, China*

^#^ These authors contributed equally to this work.

* To whom correspondence should be addressed:

1. Tongkai Chen

Science and Technology Innovation Center, Guangzhou University of Chinese Medicine, 12 Jichang Road, Guangzhou 510405, China

Tel.: +86 20 36585707

E-mail: chentongkai@gzucm.edu.cn

2. Guangtao Yu

Stomatological Hospital, School of Stomatology, Southern Medical University, Guangzhou 510280, China

E-mail: guangtao1986@smu.edu.cn

**S. Materials and methods**

S1 Materials

Bulk black phosphorus (BP) was purchased from Nanjing Xianfeng Nano Material Technology Co., Ltd. (Nanjing, China). FITC-PEG-NH_2_ and PEG-NH_2_ with a PEG molecular weight of 2000 were obtained from Xi’an Ruixi Biological Technology (Xi’an, China). Metformin (Met) was obtained from Feiyu Biological Technology Co., Ltd. (Nantong, China). N-methyl-2-pyrrolidone (NMP, 99.5%, anhydrous) was obtained from Aladdin Reagents (Shanghai, China). Sodium hydroxide (NaOH) was sourced from Shanghai Macklin Biochemical Co., Ltd. (Shanghai, China). Cell Counting Kit-8 (CCK-8) was purchased from Dojindo Laboratories (Kumamoto, Japan). Lysosome (Lyso)-Tracker was procured from MedChemExpress (Monmouth Junction, New Jersey, USA). Endoplasmic Reticulum (ER)-Tracker was obtained from Shanghai Biorbyt Biotechnology Co., Ltd. (Shanghai, China). Mitochondria (Mito)-Tracker was purchased from Thermo Fisher Scientific (Waltham, Massachusetts, USA). The Live/Dead Cell Double Staining Kit was purchased from BioLegend (San Diego, California, USA). Antibodies against Ki67, CD31, and 4-Hydroxy-2-nonenal (4-HNE) were all purchased from Abcam (Cambridge, UK). Antibodies against proliferating cell nuclear antigen (PCNA) were purchased from Cell Signaling Technology (Beverly, Massachusetts, USA), while the anti-VEGF antibody was purchased from Beijing Biosynthesis Biotechnology Co., Ltd. (Beijing, China). The Malondialdehyde Assay (MDA) Kit was procured from Beyotime Biotechnology Co., Ltd. (Shanghai, China). Hematoxylin and eosin (H&E) staining reagents were obtained from Beijing Lan Jie Ke Technology Co., Ltd. (Beijing, China). Nile red (NR) was purchased from Sigma-Aldrich (St. Louis, USA). The Mitochondrial Membrane Potential Assay Kit (JC-1) was purchased from the Beyotime Institute of Biotechnology (Shanghai, China).

S2 Animals

C57BL/6 male mice, weighing 18-22 g, were purchased from Zhejiang Vital River Laboratory Animal Technology Co., Ltd. Before animal experiments were initiated, all the mice were reared in an SPF environment under a normal light-dark rhythm of 12 h each, a temperature of 25 ± 2°C, and relative humidity of 55 ± 5%. To ensure the ethical treatment of experimental animals, all procedures were approved by the animal ethics committee of Guangzhou University of Chinese Medicine and conformed to institutional guidelines (NO. 20220510011).

S3 Preparation and characterization of black phosphorus nanosheets (BP NSs)

BP NSs were prepared using a modified liquid exfoliation technique [1]. Briefly, bulk BP was dispersed in NMP at an initial concentration of 1 mg/mL. The dispersion was sonicated in an ice bath for 24 h using an ultrasonic cell disruption system (300 W, 4 s on and 6 s off) to obtain a brown suspension. This brown suspension was centrifuged at 2000 × g for 20 min to remove unexfoliated bulk BP. Then, the supernatant containing BP NSs was collected and re-centrifuged at 2000 rpm for 20 min. The resulting precipitate provided the BP NSs.

The prepared BP NSs were examined using scanning electron microscopy (SEM), transmission electron microscopy (TEM), and atomic force microscopy (AFM). The crystal structure of BP NSs was analyzed using X-ray powder diffractometry (XRD) and X-ray photoelectron spectroscopy (XPS). The particle size, polydispersity index (PDI), and zeta potential of BP NSs were measured using a particle sizing system (PSS NICOMP 380ZLS, USA).

S4 Determination of drug loading efficiency

The drug loading of BP-PEG-Met was calculated by determining the amount of free Met using ultrafiltration technique. 0.5 mL of BP-PEG-Met was placed in the upper chamber of a centrifuge tube matched with an ultrafilter (MWCO 10 kDa), and centrifuged for 30 min at 3000 × g with the temperature of 4 °C. The ultra-filtrate containing the free Met was determined using high performance liquid chromatography (HPLC). The drug loading efficiency (DLE) is calculated using the following formula:

Drug loading efficiency (DLE) (%)= $\frac{\text{W}\text{eig}\text{h}\text{t of Met in BP-PEG-Met}}{\text{T}\text{otal weig}\text{h}\text{t of}\text{ }\text{BP}\text{-}\text{PEG}\text{-}\text{Met}}$×100%

S5 Preparation and characterization of BP-PEG-Met

To improve the biocompatibility of BP NSs, we modified them using PEG-NH_2_. Briefly, BP NSs and PEG-NH_2_ were mixed in deoxygenated ultrapure water at a mass ratio of 1:5 and then stirred in the dark for 4 h at room temperature. Subsequently, the mixture was centrifuged at 2000 × g for 20 min, and the precipitate was collected and washed thrice with deoxygenated ultra-pure water to obtain BP-PEG. In the next step, BP-PEG was mixed with Met in deoxygenated ultrapure water at a mass ratio of 1:20, and the mixture was stirred at 25 ℃ for 6 h. It was then centrifuged at 2000 × g for 10 min to remove unloaded Met. Finally, BP-PEG-Met was obtained.

TEM was used to observe BP-PEG-Met. A particle sizing system (PSS NICOMP 380ZLS, USA) was employed to measure the particle size, PDI, and zeta potential of BP-PEG and BP-PEG-Met. An ultraviolet–visible (UV-Vis) spectrophotometer was used to measure the optical density (O.D.) of BP-PEG-Met at 233 nm. In addition, the changes in the particle size, PDI, and UV-Vis spectra of BP NSs and BP-PEG-Met during storage were measured on days 1, 3, 5, and 7 to evaluate the stability of BP-PEG-Met.

S6 *In vitro* drug release study

Dialysis method was used to evaluate the in vitro drug release profiles of Met and BP-PEG-Met. Met and BP-PEG-Met suspensions (5 mL) were placed inside a dialysis bag (molecular weight cutoff of 12 kDa) and kept in a beaker containing phosphate-buffered saline (PBS, 45 mL, pH 7.4) at 37.0 ± 1.0 ℃ in an incubator shaker with continuous stirring at 120 rpm. At 0.5, 1, 2, 3, 4, 6, 8, 12, and 24 h, sample aliquots (5 mL) were taken from the medium for analysis and replaced with an equivalent amount of PBS. The amount of Met and BP-PEG-Met in these samples was examined via UV-Vis spectrophotometric measurements at 233 nm. The O.D values obtained at different time points were substituted into the standard curve to obtain drug concentrations at specific time points. Then, the following formula was used to calculate cumulative drug release:

Q_n_=[C_n_V+$\sum_{i=1}^{n-1} C$_i_V_i_]

where C_n_ represents the drug concentration measured at the nth sampling point, C_i_ represents the concentration measured in each sample, V represents the volume of the medium, V_i_ represents the volume of each sample, and Q_n_ represents the cumulative amount of the drug.

S7 *In vitro* transdermal experiments and skin retention of Met and BP-PEG-Met

Sprague–Dawley (SD) rats were sacrificed, and their dorsal skin was obtained. Vertical Franz diffusion cells with an effective diffusion area of 1.766 cm^2^ were used for the permeation study. The receptor compartment was filled with 14 mL saline, kept in a water bath at 32 ℃, and stirred at 300 rpm. The skin samples were placed between the donor cell and receptor cell, with the stratum corneum (SC) facing the former. The Met solution and BP-PEG-Met suspension were placed in the donor cell, which was in direct contact with the SC. At predetermined time points (15 min, 30 min, 1 h, 2 h, 4 h, 7 h, and 8 h), 1 mL of saline was withdrawn from the receptor cell, and the same volume of fresh saline was injected into the receptor cell. The withdrawn samples were examined using UV-Vis spectrophotometry at 233 nm. Then, the standard curve of Met was employed to calculate the cumulative permeation per unit area Q_n_.

Q_n_=[C_n_V+$\sum_{i=1}^{n-1} C$_i_V_i_]/S,

where C_n_ is the concentration of Met in the receptor medium at the nth sampling point, C_i_ is the concentration of Met in the receptor medium at the ith time point, V is the volume of the receptor cell, V_i_ is the volume of the sample, S is the effective permeation area of the diffusion cell, and Q_n_ is the cumulative permeation amount per unit area.

Additionally, after a 24 h transdermal experiment, rats were sacrificed and their skin was collected. Any drug remaining on the surface of the skin was washed off, and the skin was cut into small pieces and placed in a 50 mL centrifuge tube. Then, 20 mL of 50% ethanol solution was added, and the tubes were allowed to stand for 30 min before 30 min of sonication. Subsequently, the tubes were centrifuged at 4000 × g and 4°C for 10 min. The supernatant was filtered through a 0.22 μm membrane, and its absorbance value was measured at 233 nm using a UV-vis spectrophotometer. Then, the standard curve of Met was employed to calculate the retention of Met in the skin.

S8 *In vitro* antioxidant activity of BP NSs and BP-PEG-Met

S8.1 ABTS assay

An ABTS working solution was prepared according to the manufacturer’s instructions. Different concentrations of BP NSs and BP-PEG-Met were mixed with the ABTS working solution and incubated for 10 min. Then, the absorbance of the ABTS working solution was measured at 734 nm using a UV-Vis spectrophotometer.

S8.2 DPPH assay

A stock solution of DPPH in methanol was prepared and kept in the dark for 60 min. Then, its absorbance was measured at 517 nm (i.e., the maximum wavelength of absorption for DPPH). Different concentrations of BP NSs and BP-PEG-Met were mixed with the DPPH solution and kept in the dark for 30 min. Then, the absorbance of the DPPH solution at 517 nm was measured using a UV-Vis spectrophotometer.

S8.3 •OH assay

Hydroxyl radicals (•OH) generated by the Fenton reaction can degrade Methylene blue (MB). Thus, the absorbance of residual MB was examined to evaluate the •OH scavenging ability of different concentrations of BP NSs and BP-PEG-Met. After incubation in the dark for 5 min, the absorbance of the MB supernatant was measured at 652 nm using a UV-Vis spectrophotometer.

S8.4 •O_2_^-^ assay

•O_2_^-^ was generated by adding pyrogallic to Tris-HCl, and this solution was mixed with different concentrations of BP NSs and BP-Met. After incubation in the dark for 30 min, the absorbance of the solution was measured at 325 nm using a UV-Vis spectrophotometer.

S9 Cellular experiments

S9.1 Cytotoxicity assay

Human skin fibroblast cells (HSFCs) were cultured in DMEM/F12 containing 10% fetal bovine serum (FBS) and 1% penicillin–streptomycin under 5% CO_2_. The cytotoxicity of BP-PEG-Met and Met were evaluated based on cell proliferation, which was examined using the CCK-8 kit. First, HSFCs (1×10^3^ cells/well) were seeded in a 96-well plate for 24 h. Except for the Control group, the other groups were treated with Met (2.5, 5, 10, 20, and 40 mM) and BP-PEG-Met (1.25, 2.5, 5, 10, and 20 μg/mL). After co-incubation for 24 h, the CCK-8 working solution was added to each well and incubated for 20 min. Then, the absorbance of each well was measured at a wavelength of 450 nm using a microplate reader.

In addition, Annexin V/propidium iodide (PI) staining was used to evaluate the cytotoxicity of BP. HSFCs (1×10^6^ cells/well) were seeded in a 6-well plate for 24 h. Cells were then treated with different concentrations of BP NSs (1.25, 2.5, 5, 10, and 20 μg/mL) for 24 h. Following that, cells were harvested, washed twice with cold PBS, and resuspended in 100 μL binding buffer. Finally, Annexin V-FITC (5 μL) and PI (10 μL) were added, and the cell apoptosis rate was analyzed by flow cytometry.

Additionally, cytotoxicity was also evaluated using the Live/Dead Assay. HSFCs were seeded into a 12-well plate at a density of 1×10^5^ cells/well and incubated for 24 h. Then, the medium was replaced with fresh medium containing BP NSs (10 μg/mL), Met (20 mM), or BP-PEG-Met (10 μg/mL) for 24 h. At the end of the incubation period, calcein-AM/propidium iodide dye solution was added, and the cells were incubated for 30 min in the dark. Then, cells were observed using confocal laser scanning microscopy (CLSM) under the green (492 nm) and red (545 nm) fluorescence channels.

S9.2 Cellular uptake of BP NSs

HSFCs were seeded in a 12-well plate at a density of 1×10^5^ cells/well and incubated for 24 h. Then, FITC-labeled BP NSs (BP-FITC, 50 μg/mL) was added and the cells were treated for 1, 2, and 4 h. The cells were subsequently washed with PBS and incubated with DAPI for 8 min. Finally, they were fixed with 4% paraformaldehyde for 10 min.

In addition, the uptake of BP NSs by HSFCs was also evaluated under different concentrations. HSFCs were seeded in a 12-well plate at a density of 1×10^5^ cells/well and incubated for 24 h. Then, different concentrations of BP-FITC (12.5, 25, and 50 μg/mL) were added, and the cells were treated for 4 h. The cells were subsequently washed with PBS and incubated with DAPI for 8 min. Finally, they were fixed with 4% paraformaldehyde for 10 min.

The method used for preparing BP-FITC was based on previous literature but was modified slightly [2]. BP NSs and FITC-PEG-NH_2_ were mixed at a feeding ratio of 1:5. The mixture was stirred overnight at 600 rpm in the dark. Then, it was centrifuged, and the unloaded FITC-PEG-NH_2_ in the supernatant was discarded. The precipitate was washed thrice with DMEM/F12, and BP-FITC was obtained.

S9.3 Cellular internalization mechanism of BP NSs

A slightly modified version of a previously reported protocol was employed to explore the internalization pathways of BP NSs in HSFCs [3]. First, the cells were seeded in a 12-well plate at a density of 1×10^5^ cells/well and incubated for 24 h. Then, the medium was replaced with fresh medium containing chlorpromazine (CPZ, 10 μg/mL), hypertonic sucrose (HS, 0.2 M), ethylisopropylamiloride (EIPA, 10 μg/mL), or Methyl-β-cyclodextrin (MβCD, 5 mM) to inhibit internalization via clathrin, caveolin, and micropinocytosis. The cells were then treated with Nile red-labeled BP NSs (BP-NR, 50 μg/mL) for 4 h in the dark. The group treated with BP-NR in the absence of inhibitors served as the Control.

BP-NR was prepared as described previously, with slight modifications [2]. BP NSs and NR were mixed at a feeding ratio of 1:5, and the mixture was stirred overnight in the dark at 600 rpm. Subsequently, the mixture was centrifuged, and the unloaded NR in the supernatant was discarded. The precipitate was washed thrice with DMEM/F12 to obtain BP-NR.

S9.4 Cellular co-localization of BP NSs

The HSFCs were seeded in a 12-well plate at a density of 1×10^5^ cells/well and incubated for 24 h. Then, the cells were co-incubated with DMEM containing Mito-Tracker, ER-Tracker, or Lyso-Tracker at 37 °C for 30 min. Subsequently, the cells were co-incubated with medium containing BP-FITC (50 μg/mL) for 4 h. After that, the cells were washed with PBS and incubated with DAPI in the dark for 10 min to stain the nuclei. The cells were again washed with PBS, fixed with 4% paraformaldehyde for 15 min, and washed with PBS once more. Finally, the cells were observed using CLSM.

S9.5 Detection of mitochondrial membrane potential

HSFCs were seeded in a 12-well plate at a density of 1×10^5^ cells/well. When the cells reached 90% confluence, they were pretreated with 20 mM Met, BP-PEG-Met, or 10 μM carbonyl cyanide m-chloro phenylhydrazone (CCCP) for 4 h. Notably, the Control group was left untreated. After staining with JC-1, the green fluorescence signals of JC-1 monomers and the red fluorescence of JC-1 aggregates were observed under a confocal microscope. The kit was used in accordance with the manufacturer’s instructions.

S9.6 ROS-scavenging activity assay

The ROS-scavenging activity of BP NSs was examined as described previously, with slight modifications [4]. HSFCs were seeded in 12-well plates at a density of 1×10^5^ cells/well and incubated for 24 h. Then, the cells were incubated with H_2_O_2_ (900 μM) for 30 min. Following this, fresh medium containing various concentrations of BP NSs (0, 5, 10, 20, 40, and 80 μg/mL) was added for 4 h. At the end of the incubation period, the cells were washed with PBS and incubated with a DCFH-DA working solution for 20 min at 37 ℃. The cells were fixed with 4% paraformaldehyde for 10 min before incubation with DAPI for nuclear staining. Subsequently, the fluorescence intensity of DCFH-DA in HSFCs was detected with flow cytometry. Meanwhile, the cell viability was determined by the CCK-8 assay.

S10 *In vivo* treatment of AGA

The hair regeneration capabilities of BP-PEG-Met were evaluated in male C57BL/6 mice (7 weeks old). Animals were acclimatized to the experimental environment for 1 week. A 1-cm^2^ region (1 cm×1 cm) of hair in the telogen phase was gently shaved from the dorsal skin of the 8-week-old mice using an electric hair clipper and depilated with hair removal cream. The mice were randomly divided into five groups: the model group, the minoxidil (MXD) group, the Met group, the BP-PEG-Met group, and the control group. The AGA mouse model was established based on previously reported protocols, with slight modifications [5]. Briefly, a TS solution (5 mg/mL, 200 μL) was prepared in ethanol (50%, v/v). Except for the control group, all other groups received topical TS solution application on the depilated area daily for 28 consecutive days. Meanwhile, the MXD, Met, and BP-PEG-Met groups received topical applications of MXD (250 mg/kg), Met (2 mg/mL, 100 μL), and BP-PEG-Met (2 mg/mL, 100 μL), respectively, every other day until day 28. The control group did not receive any additional treatment during the 28-day period. Digital photographs of the skin and hair were obtained across all groups every 7 days.

S11 Evaluation of the quality of newly grown hair

On the 28th day after hair removal, the newly grown hair of the mice was shaved off and collected. The weight of this newly grown hair was measured using an analytical balance. Subsequently, five hairs were randomly selected from each group, and their length was measured using vernier calipers. Finally, the coverage rate of the newly grown hair was measured using Image J. Additionally, the surface structure of the hair was observed using SEM.

S12 Histology and immunofluorescence staining

Animals were sacrificed on day 29 for the detection of Ki67 expression, PCNA expression, and H&E staining, and for the detection of 4-HNE, CD31, and VEGF levels in the depilated area. Skin samples were first fixed using 4% formaldehyde. Following sequential dehydration, the tissues were embedded in paraffin, and 4 μm thick sections were subsequently obtained. Following deparaffinization and rehydration, the sections were stained with hematoxylin for 30 s and eosin for 1 min. H&E-stained sections were imaged using a Digital Pathology Scanner. For immunohistochemistry, the sections were incubated with anti-Ki67 and anti-PCNA antibodies to examine Ki67 and PCNA expression in the skin.

For immunofluorescence evaluations, deparaffinized and rehydrated tissue sections were further incubated with anti-4-HNE, anti-VEGF, and anti-CD31 antibodies. This was followed by incubation with the corresponding fluorescence-labeled secondary antibodies and nuclear staining with DAPI. All slides were observed and photographed using CLSM.

S13 Detection of MDA

Skin samples were collected from depilated areas at a predetermined time point. The samples were cut into small pieces, placed in EP tubes, and mixed with steel balls for homogenization at a frequency of 65 Hz for 2 min. Then, ultrasonic treatment was performed in an ice-water bath for 2 min to obtain the final homogenate. The homogenate was centrifuged at 12,000 rpm and 4 °C for 15 min. The supernatant was collected, and the content of MDA in the skin tissue was measured according to the instructions of the MDA assay kit.

S14 Statistical analysis

Data were expressed as the mean ± standard deviation (SD). ANOVA and related post hoc tests were employed to analyze the differences in means among different experimental groups. All analyses were performed using GraphPad Prism version 9.5 software. The significance thresholds were denoted as follows: Ns, non-significant (*p* > 0.05); **p* < 0.05, ***p* < 0.01, ****p* < 0.001, and *****p* < 0.0001.

**S. Results**


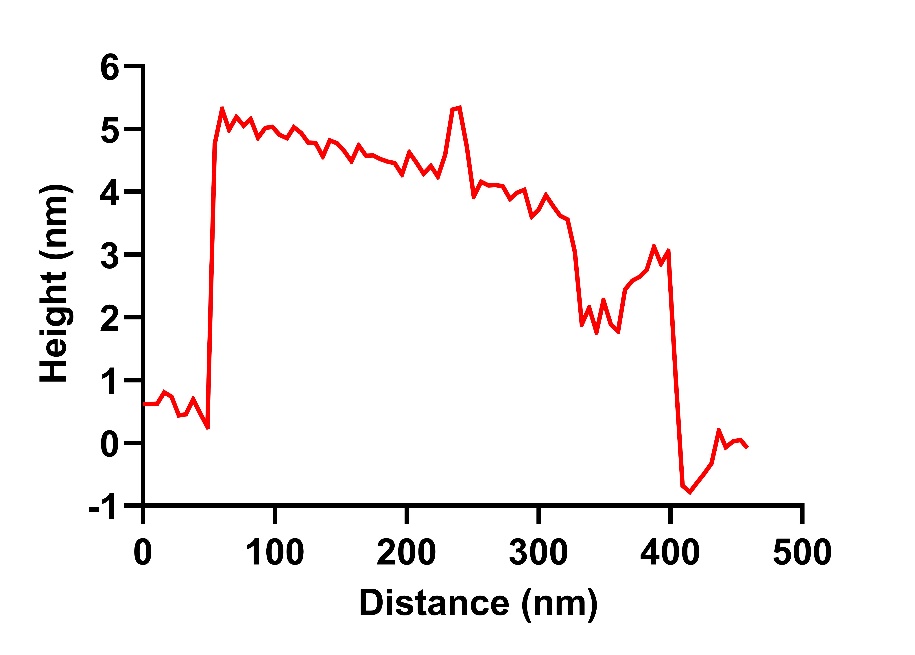


**Figure S1.** The quantification of BP in the AFM image.


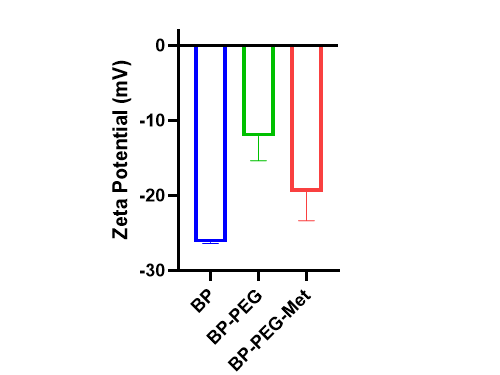


**Figure S2.** Zeta potential of BP, BP-Met, and BP-PEG-Met (n = 3).


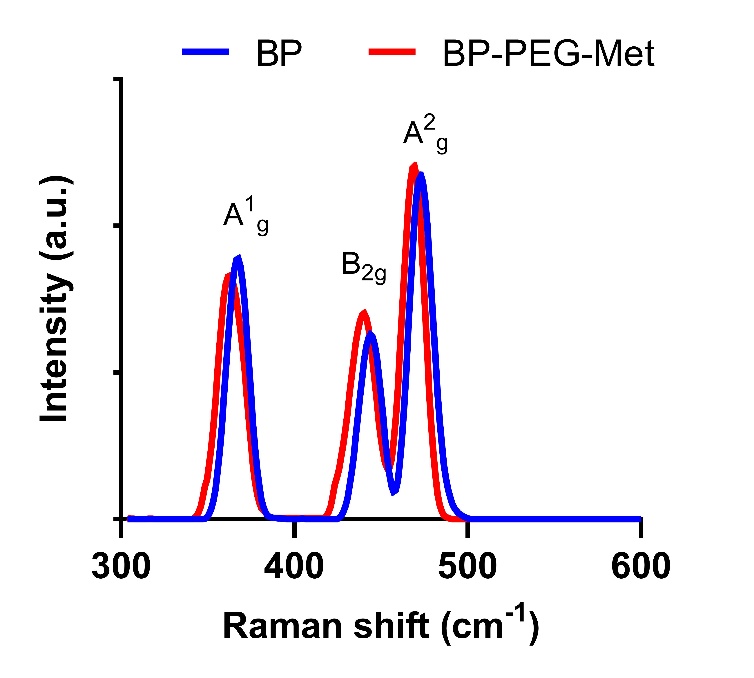


**Figure S3.** Raman spectra of BP and BP-PEG-Met.


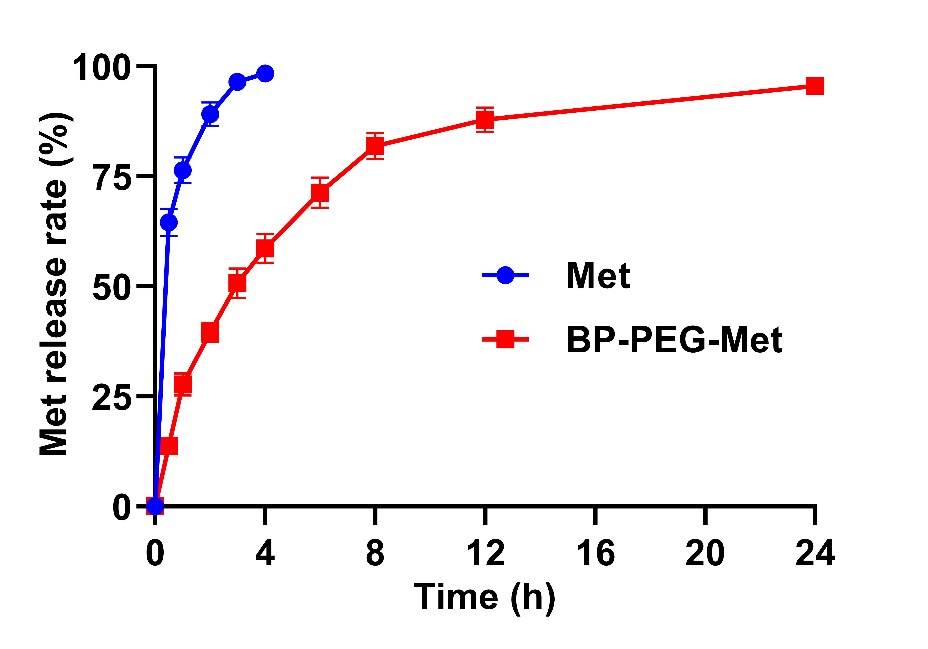


**Figure S4.** *In vitro* drug release from Met and BP-Met (n = 3).


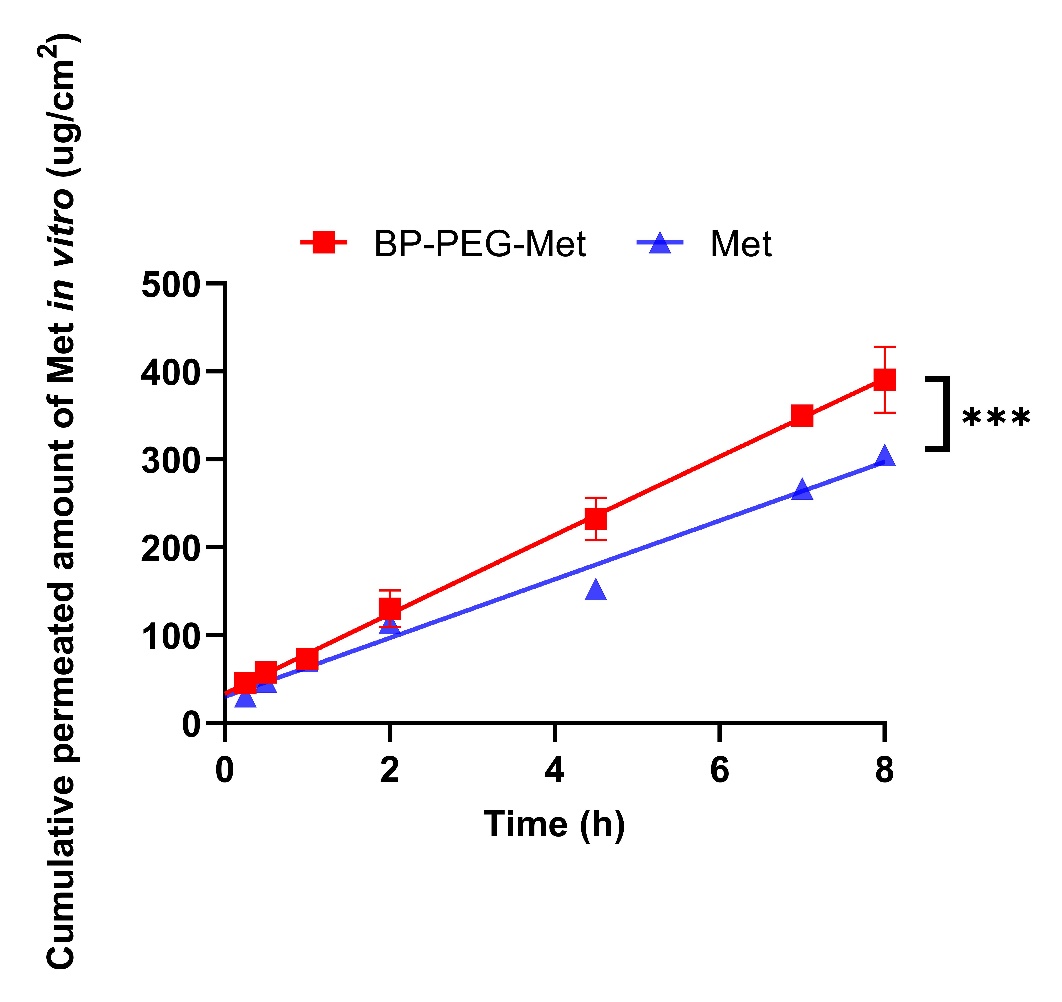


**Figure S5.** The *in vitro* skin permeation amounts of Met and BP-PEG-Met (n = 3). ****P* < 0.001 vs the Met group.


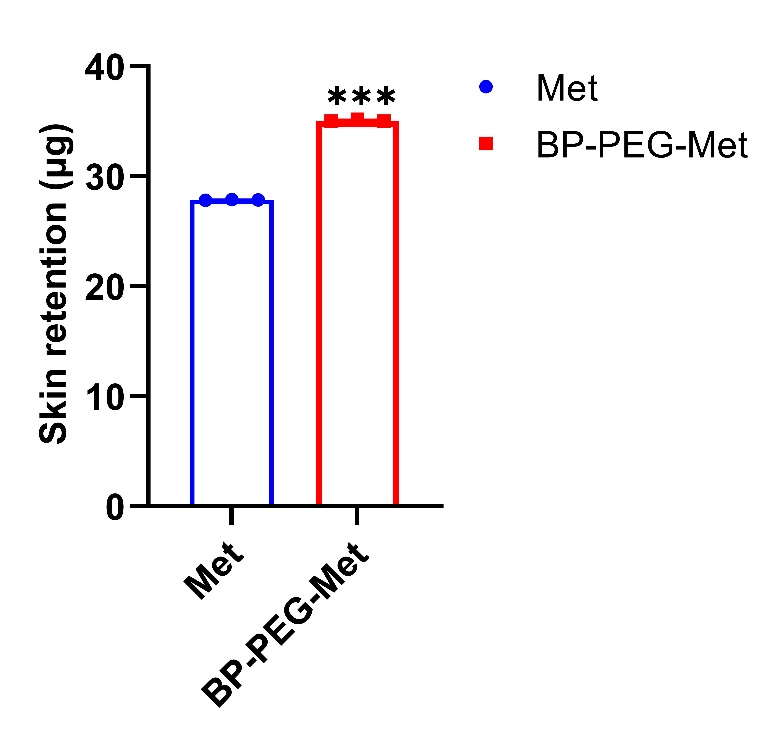


**Figure S6.** *Ex vivo* skin retention of Met after 24 h permeation from Met and BP-PEG-Met (n = 3). ****P* < 0.001 vs the Met group.


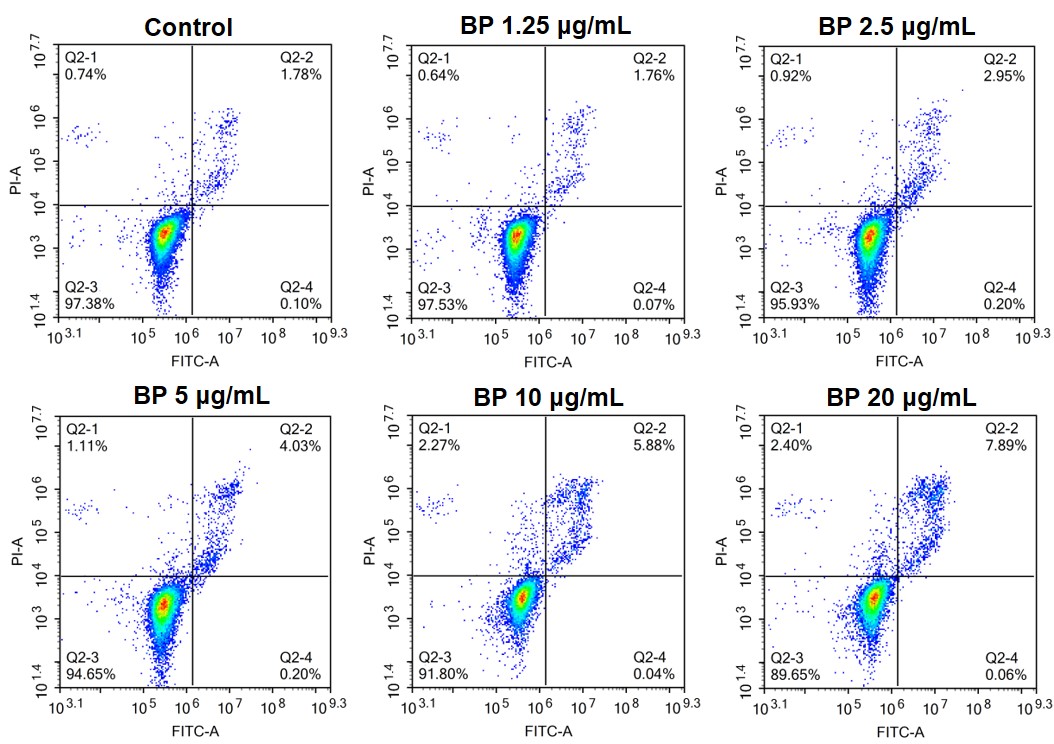


**Figure S7.** Flow cytometry was used to quantify apoptosis of cells treated with different concentrations of BP.


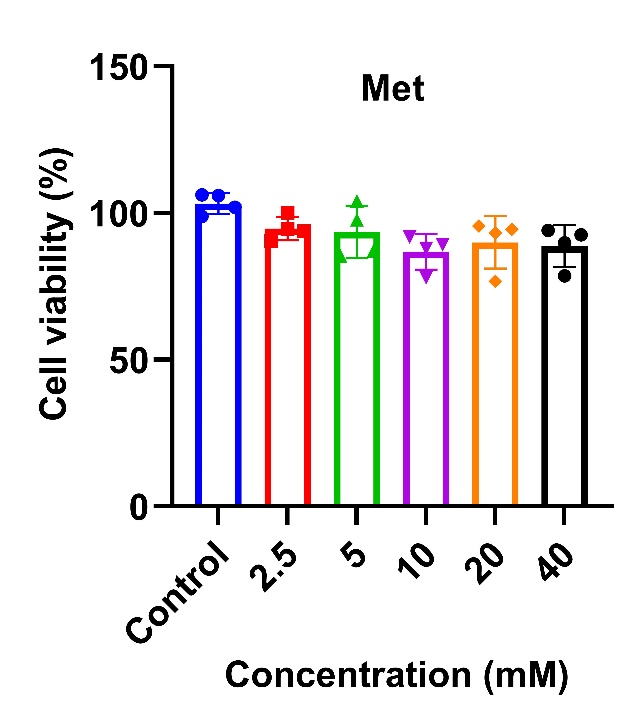


**Figure S8.** The viability of HSFCs after treatment with different concentrations of Met (n = 4).


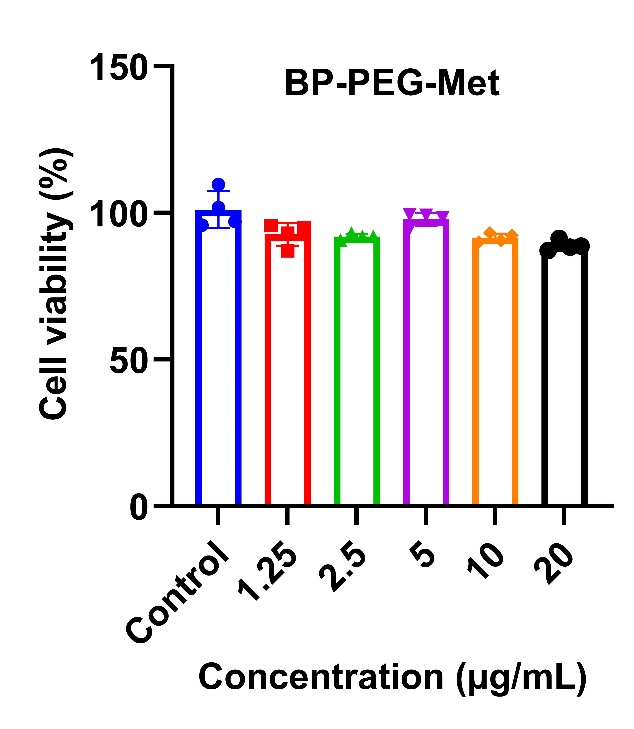


**Figure S9.** The viability of HSFCs after treatment with different concentrations of BP-PEG-Met (n = 4).


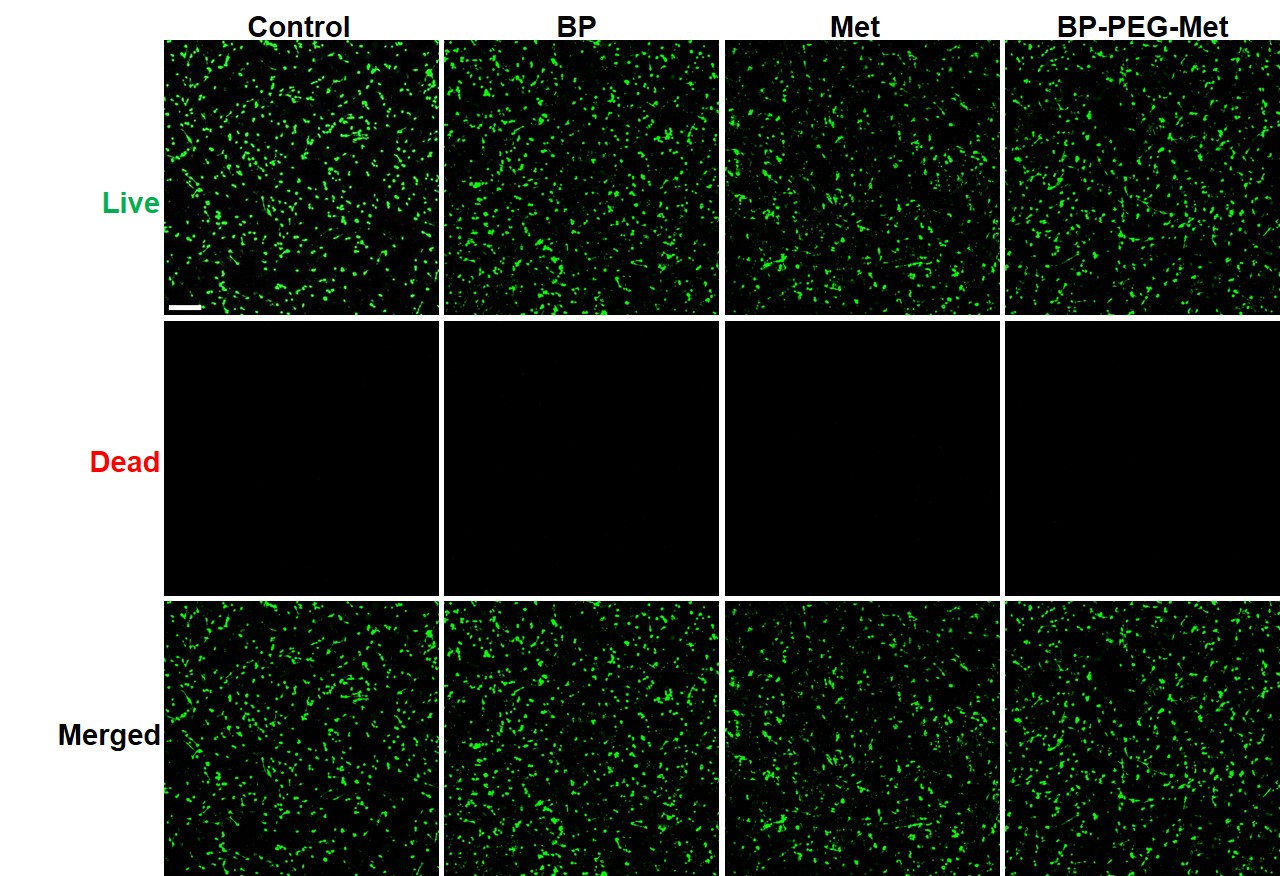


**Figure S10.** Fluorescent images of HSFCs stained by Calcein AM and Propidium Iodide (PI) after treatment with BP, Met, and BP-Met for 24 h. Scale bar: 50 μm.


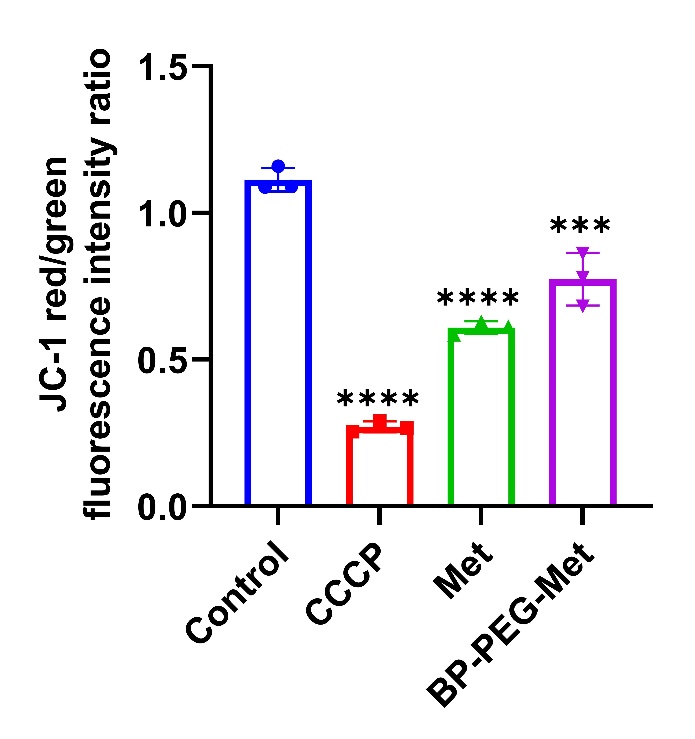


**Figure S11.** JC-1 red/green fluorescence intensity ratio (n = 3). ****p* < 0.001 and *****p* < 0.0001 vs the control group.


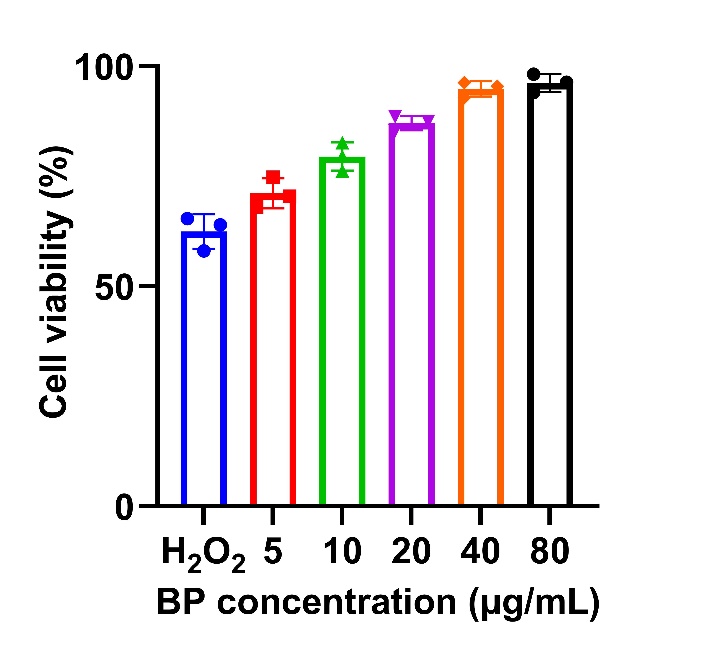


**Figure S12.** Evaluation of the protective effect of BP NSs at different concentrations on cells (n=3).


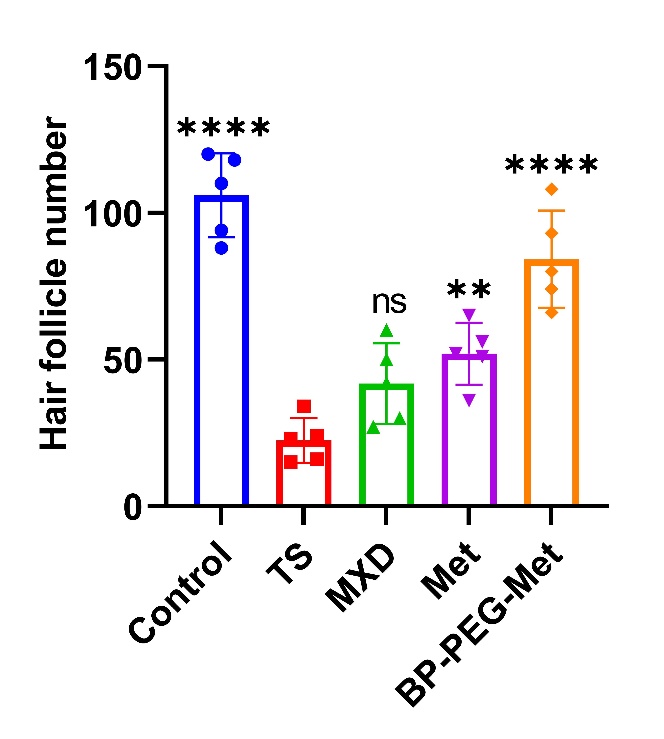


**Figure S13.** Quantification of hair follicle number (n = 5). No significance (ns, *P* > 0.05), ***p* < 0.01, and *****p* < 0.0001 *vs* the TS group.


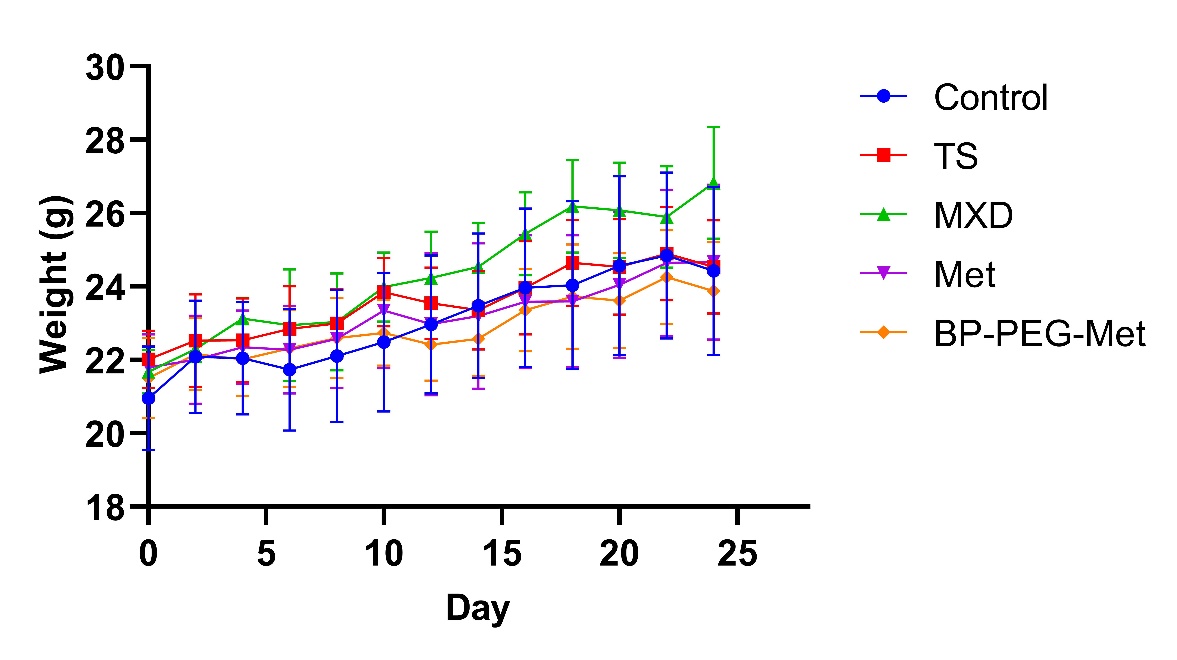


**Figure S14.** The body weight change curves of mice after different treatments (n = 5). No significance (ns, *P* > 0.05) *vs* the control group.

**Table S1** Drug loading of BP-PEG-Met and other Met-loaded carriers

| Carriers | Drug loading | References |
| --- | --- | --- |
| **BP-PEG-Met** | **83.87 ± 3.94%** |  |
| Liposome | 26.0% ± 2.8% | [6] |
| Nanoparticles | 73.2% | [7] |
| MOF(Ti) | 84.33% | [8] |
| Mn-MSN@Met-M NPs | 12.92% | [9] |
| (PA) LB-MSNs | 40% | [10] |

**Table S2** The drug release fitting equations of BP-PEG-Met

|  | Model | Equation | R^2^ |
| --- | --- | --- | --- |
| BP-PEG-Met | Zero order kinetics | Q=3.5995t+30.90648 | 0.6254 |
|  | First order kinetics | Q=93.0196 (1-e^-0.26471t^) | 0.9924 |
|  | Higuchi kinetics | Q=21.4778t^1/2^+8.66433 | 0.8956 |

**Table S3** The model fitting equations for *in vitro* skin permeation amounts of Met and BP-PEG-Met

|  | Model | Equation | R^2^ |
| --- | --- | --- | --- |
| Met | Zero order kinetics | Q=2.0365t+5.5936 | 0.8489 |
|  | First order kinetics | Q=50.8364(1-e^0.09871t^) | 0.9952 |
|  | Higuchi kinetics | Q=-6.1761t^1/2^-11.4012 | 0.9802 |
| BP-PEG-Met | Zero order kinetics | Q=2.9904t+5.4781 | 0.9313 |
|  | First order kinetics | Q=88.4890(1-e^0.06299t^) | 0.9980 |
|  | Higuchi kinetics | Q=-10.4897t^1/2^+16.1556 | 0.9913 |

**References**

[1] Yasaei P, Kumar B, Foroozan T, Wang C, Asadi M, Tuschel D, Indacochea J E, Klie R F,Salehi-Khojin A. High-quality black phosphorus atomic layers by liquid-phase exfoliation. Adv Mater 2015; 27 (11):1887-1892.

[2] Liu J, Du P, Liu T, Córdova Wong B J, Wang W, Ju H,Lei J. A black phosphorus/manganese dioxide nanoplatform: Oxygen self-supply monitoring, photodynamic therapy enhancement and feedback. Biomaterials 2019; 192:179-188.

[3] Sheng X, Zhong Y, Zeng J, Tang X, Xing J, Chi H,Zhan W. Lymphocystis Disease Virus (Iridoviridae) Enters Flounder (Paralichthys olivaceus) Gill Cells via a Caveolae-Mediated Endocytosis Mechanism Facilitated by Viral Receptors. Int J Mol Sci 2020; 21 (13):4722.

[4] Yang W, Lv Y, Wang B, Luo S, Le Y, Tang M, Zhao R, Li Y,Kong X. Polydopamine Synergizes with Quercetin Nanosystem to Reshape the Perifollicular Microenvironment for Accelerating Hair Regrowth in Androgenetic Alopecia. Nano Lett 2024; 24 (20):6174-6182.

[5] Ding Y W, Li Y, Zhang Z W, Dao J W,Wei D X. Hydrogel forming microneedles loaded with VEGF and Ritlecitinib/polyhydroxyalkanoates nanoparticles for mini-invasive androgenetic alopecia treatment. Bioact Mater 2024; 38:95-108.

[6] Xiong W, Qi L, Jiang N, Zhao Q, Chen L, Jiang X, Li Y, Zhou Z,Shen J. Metformin Liposome-Mediated PD-L1 Downregulation for Amplifying the Photodynamic Immunotherapy Efficacy. ACS Appl Mater Interfaces 2021; 13 (7):8026-8041.

[7] Yu Y, Chen J, Liu S,Cheng D. ROS-responsive organosilica nanocarrier for the targeted delivery of metformin against cancer with the synergistic effect of hypoglycemia. J Mater Chem B 2021; 9 (30):6044-6055.

[8] Tian M, Fan C, Ge R, Liu G, Su L,Dong H. Visible light-activated Ti-MOF loaded metformin-hydrogel composite dressing for accelerated chronic diabetic wound healing. Chem. Eng. J. 2025; 506:160179.

[9] Dou Y, Zheng J, Kang J, Wang L, Huang D, Liu Y, He C, Lin C, Lu C, Wu D, et al. Mesoporous manganese nanocarrier target delivery metformin for the co-activation STING pathway to overcome immunotherapy resistance. iScience 2024; 27 (7):110150.

[10] Banala V T, Sharma S, Barnwal P, Urandur S, Shukla R P, Ahmad N, Mittapelly N, Pandey G, Dwivedi M, Kalleti N, et al. Synchronized Ratiometric Codelivery of Metformin and Topotecan through Engineered Nanocarrier Facilitates In Vivo Synergistic Precision Levels at Tumor Site. Adv Healthc Mater 2018; 7 (19):e1800300.
